# Future Orientation in Adolescents: Development and the Roles of Parenting in Different Income Countries

**DOI:** 10.1007/s10964-025-02288-4

**Published:** 2025-11-25

**Authors:** Maria Chiara Basilici, Laura Gorla, Jennifer E. Lansford, Liane P Alampay, Suha M Al-Hassan, Dario Bacchini, Marc H. Bornstein, Lei Chang, Kirby Deater-Deckard, Laura Di Giunta, Kenneth A. Dodge, Sevtap Gurdal, Daranee Junla, Paul Oburu, Concetta Pastorelli, Ann T. Skinner, Emma Sorbring, Laurence Steinberg, Liliana Maria Uribe Tirado, Saengduean Yotanyamaneewong, Qin Liu, Qian Long

**Affiliations:** 1 University of Florence, Florence, Italy; 2 Duke University, Durham, North Carolina, USA; 3 Ateneo de Manila University, Quezon, Philippines; 4 Abu Dhabi Early Childhood Authority, Abu Dhabi, United Arab Emirates; 5 University of Naples“Federico II,”, Naples, Italy; 6 Eunice Kennedy Shriver National Institute of Child Health and Human Development USA, Institute for Fiscal Studies, United Kingdom & UNICEF, USA; 7 University of Macau, Avenida da Universidade, Taipa, Macau, China; 8 University of Massachusetts Amherst, Amherst, Massachusetts, USA; 9 Università di Roma “La Sapienza,”, Rome, Italy; 10 University West, Throllhattan, Sweden; 11 Chiang Mai University, Chiang Mai, Thailand; 12 Maseno University, Maseno, Kenya; 13 Temple University, USA & King Abdulaziz University, Jeddah, Saudi Arabia; 14 Universidad de San Buenaventura, Medellin, Colômbia; 15 Chongqing Medical University, Chongqing, China; 16 Duke Kunshan University, Suzhou, China

**Keywords:** Future orientation, Adolescence, Parental monitoring, Family obligations, Conformity, Income

## Abstract

Future orientation – the ability to envision and plan for the future – is a crucial task during adolescence. However, little is known about how adolescents’ future orientation develops, how it is influenced by parenting dimensions, and how it varies across countries with different income levels and cultural values. This longitudinal study addresses this gap by exploring how parents’ monitoring, family obligations, individualism, collectivism, and conformity influence future orientation. The sample is composed of 1,086 adolescents (50.5% females) at ages 10, 14, 17, and 20 (mean age at study time 1 = 10.8, *SD* = 0.69) and their 1,071 parents (85% mothers; mean age at study time 1 = 39.4, *SD* = 6.8), divided into high-income (Italy, the United States), upper-middle-income (Colombia, Thailand), and lower-middle-income countries (Jordan, Kenya, the Philippines). Full information maximum likelihood multigroup latent growth curve models revealed that adolescents’ future orientation trajectories are nonlinear, that family obligations and conformity values are the parenting dimensions more strongly related to future orientation, and differences across broader economic and cultural contexts. This study clarifies the role of parental monitoring, family obligations, individualism, collectivism, and conformity values in shaping adolescents’ future orientation across cultures, highlighting the importance of responsive parenting and balanced family obligations for family-focused policies and programs.

**Supplementary Information:**

The online version contains supplementary material available at 10.1007/s10964-025-02288-4.

## Introduction

Adolescence is a crucial development period characterized by profound cognitive, emotional, and social changes (Seginer, 2005). Among the key developmental tasks of adolescence, identity formation and long-term decision-making are particularly critical and are supported by future orientation (Seginer, 2005). Future orientation is a core psychological construct that plays a crucial role in adaptive development and positive life outcomes (Zhang et al., 2013). However, longitudinal research examining how future orientation develops across different contexts is limited. In particular, few studies have examined the role of parenting, family dynamics, and broader socioeconomic and cultural environments in shaping adolescents’ future orientation. The present study addresses this gap by examining how dimensions of parenting (including parental monitoring, family obligations, individualism vs. collectivism, and conformity values) predict future orientation during adolescence across seven countries with varying income levels and cultural orientations.

### Future Orientation

Future orientation is defined as individuals’ ability to envision, plan for, and invest in their future, and plays a crucial role in shaping adolescent development (Seginer, 2009). Future orientation shapes cognition, emotion, and behavior and becomes particularly salient during key developmental transitions such as adolescence. Future orientation facilitates goal setting, planning, and sustained commitment (Seginer, 2005) and is consistently linked to positive outcomes in life, including greater well-being (Zhang et al., 2013), academic success (Adelabu, 2008), career planning (Poletti et al., 2023), and diminished engagement in risky behaviors and substance use (Cui et al., 2020). Gaining a deeper understanding of the development of future orientation is essential for promoting adolescents’ well-being and supporting their capacity to make conscious life choices. Yet, empirical research in this area remains limited.

### Development of Future Orientation in Adolescence

Among the key issues in understanding future orientation, developmental stages—particularly during adolescence—have been a primary focus of research. Studies conducted in the 1980s and 1990s consistently found that future orientation tends to increase across adolescence (Furby & Beyth-Marom, 1992; Greene, 1986). Steinberg et al. (2009) corroborated these findings, showing that younger adolescents report lower levels of future orientation compared to older adolescents, particularly those aged 16 and above. These contributions were primarily based on cross-sectional designs, limiting the ability to capture developmental changes over time. Similarly, evidence regarding gender differences remains inconsistent across studies (Greene & DeBacker, 2004; Seginer & Lilach, 2004). Moreover, as contexts, societal expectations, and historical events have evolved, the factors shaping adolescents’ future orientation have also changed. For instance, a systematic review revealed the negative impact of collective stressful events (e.g., the COVID-19 pandemic) on individuals’ future orientation (Basilici et al., 2025). In consequence, longitudinal studies contribute to a better understanding of how future orientation unfolds in adolescence, and contemporary studies would update that understanding, and unpacking influences of contextual factors would broaden that understanding.

Many factors influence how adolescents envision, plan for, and pursue their future goals. The present study examined the impacts of parent- and family-related factors and how their influences shift across developmental stages. Given the profound psychological and social changes that characterize adolescence—such as the development of advanced cognitive skills (Scott & Saginak, 2016), the emergence of the ability to envision future selves (Trempala & Malmberg, 2002), and the formation of future expectations (Iovu et al., 2018)— it is crucial to analyze the potential associations between experiences in pre-adolescence and the developmental trajectory of future orientation. This developmental transition suggests that the effects of parenting may vary with age—a temporal dynamic that has received limited attention.

### Dimensions of Parenting Influencing Adolescent Future Orientation

Parenting shapes adolescent future orientation (Mahajna, 2025), and diverse dimensions of parenting might make separate contributions. Among these, monitoring plays a crucial role in fostering autonomy, planning skills, and a sense of agency—core components of future orientation. Balanced monitoring, typical of supportive parenting, provides guidance while respecting adolescents’ growing need for independence (Lin, 2023). In contrast, strict control and limited autonomy may hinder motivation and self-directed planning (Liu et al., 2024). Minimal parental monitoring and unclear boundaries have been associated with externalizing behaviors that can disrupt long-term goal setting (Barber et al., 2005). Intertwined with parental monitoring practices are also family obligations expectations, defined as the sense of duty to support family members and consider their needs when making personal decisions (Fuligni et al., 1999). Although family obligation expectations remain an underexplored factor, they may significantly influence adolescents’ future orientation. These expectations help shape adolescents’ ability to balance autonomy with relatedness, encouraging them to reflect on the broader social implications of their actions (Fuligni et al., 1999). In this way, family obligations may play a meaningful role in the development of future orientation (O’Connor et al., 1996). Because parents’ cultural orientation affects various aspects of child development (Gorla et al., 2024), it may also play a key role in adolescents’ future orientation. Specifically, the distinction between individualism and collectivism is relevant, as these cultural tendencies can either support or limit different dimensions of future-oriented development (Tamis-LeMonda et al., 2008). For instance, parents with a more individualistic orientation tend to promote autonomy, independence, and the pursuit of personal goals, which may support the development of a stronger future orientation in their children (Lee et al., 2010). In contrast, parents who endorse a collectivistic orientation often emphasize interdependence and a sense of duty toward the well-being of the group (Lee et al., 2010), potentially leading to a reduced emphasis on individual future planning. Beyond cultural orientation, it is also important to consider parents’ conformity values—that is, the extent to which mothers and fathers align their parenting practices with prevailing cultural norms. Conformity-oriented parents tend to emphasize obedience and respect for authority (Suizzo et al., 2019), shaping children’s orientation toward communal rather than agentic goals (Cheng et al., 2013). It is plausible that these socialization patterns influence adolescents’ future orientation by shaping the kinds of goals they prioritize and the ways they plan for the future.

Mothers and fathers parent adolescents differently and so may have different influences over the development of adolescents’ future orientation (Yaffe, 2023). A systematic review aimed at synthesizing studies containing significant findings on differences between mothers and fathers in parenting styles and practices found that mothers tend to be generally more nurturing and responsive, while also being more behaviorally controlling, often adopting an authoritative style (Yaffe, 2023). Fathers, in contrast, tend to exhibit an authoritarian approach (Yaffe, 2023). These patterns were observed across studies conducted in 15 countries and appear consistent regardless of adolescent gender and age. The studies included in the review relied on reports from both parents and children, providing a more comprehensive view of family dynamics (Yaffe, 2023). Therefore, in examining parenting dimensions in relation to the development of future orientation across adolescence, it is crucial to move beyond viewing parenting as homogeneous but instead acknowledge that maternal and paternal influences should be analyzed separately.

### Socioeconomic and Cultural Contexts of Future Orientation

The development of individuals’ future orientation is also shaped by the socioeconomic and cultural contexts in which individuals live (Fakkel et al., 2023), but the nature of this relation is debated. According to life history theory, people with limited resources prioritize short-term survival over long-term planning, whereas higher socioeconomic status fosters a future-oriented mindset and supports long-term goal pursuit (Chang & Lu, 2025). Consistent with life history theory, a meta-analysis showed a small but significant positive correlation between socioeconomic status and future orientation (Song & Sun, 2025). However, an alternative view posits that scarcity can enhance planning, as limited resources may prompt individuals to be more strategic and deliberate (Mullainathan & Shafir, 2013). In contrast, individuals with greater means may feel less urgency to plan ahead (Park et al., 2024). Both perspectives, however, focus mainly on the individual or family level, overlooking influences of national context and fail to recognize that a country’s economic standing shapes public resources, institutional support, and opportunity structures, which are dimensions that strongly affect how people envision their futures.

In addition to material resources, individuals are also shaped by the cultural values of the society they live in – values that are transmitted within families and across generations (Ali & Asif, 2023). These cultural frameworks influence parental beliefs and practices, shaping understanding about autonomy, responsibility, and long-term goals (Ali & Asif, 2023). For instance, adolescents from Asian and Latin American backgrounds often report stronger family obligations than their European peers, reflecting cultural norms that emphasize interdependence and respect for the family (Fuligni et al., 1999). Cultural values also underpin the concept of parental conformity, which reflects the extent to which parents align their behaviors with culturally defined expectations. Cultural context shapes the parenting practices of both mothers and fathers, including the degree to which they conform to culturally prescribed roles (Gorla et al., 2024). Parents’ cultural orientation plays a key role in shaping adolescents’ future orientation within the family context, and these influences are deeply embedded in the broader cultural values of the society in which families live: individualistic cultures tend to promote independence and personal achievement, whereas collectivistic cultures emphasize loyalty, family cohesion, and shared responsibility (Oh et al., 2020). Therefore, a comprehensive understanding of how future orientation develops requires attention to individual and family-level factors and to broader socioeconomic conditions and cultural aspects of the country where individuals live. Little or no research has investigated all these factors together.

## Current Study

Longitudinal research examining adolescents’ future orientation across diverse contexts remains particularly limited, and few studies have explored the role of parenting, family dynamics, and broader socioeconomic and cultural environments in shaping it. In this multivariate theoretical framework, the present study explores the development of adolescent future orientation and how dimensions of parenting (including parental monitoring, family obligations, individualism vs. collectivism, and conformity values) relate to future orientation during adolescence across countries with varying income levels (i.e., high, upper-middle, and lower-middle income). Future orientation is expected to increase from early to late adolescence (Hypothesis 1). Adolescents from higher-income countries are expected to report greater future orientation than their peers from upper-middle and lower-middle income countries (Hypothesis 2). Parental monitoring during pre-adolescence is expected to be negatively associated with the development of adolescents’ future orientation (Hypothesis 3). A strong sense of family obligation during pre-adolescence is expected to predict adolescents’ future orientation positively (Hypothesis 4). This association is hypothesized to be moderated by age, such that the effect is stronger in early adolescence and weaker in later adolescence. Parents’ cultural orientation during pre-adolescence is expected to influence adolescents’ future orientation (Hypothesis 5). This association is hypothesized to be moderated by country income level, such that individualistic orientation exerts a stronger positive effect on future orientation in high-income countries compared to the other income countries, whereas collectivistic orientation is expected to have a weaker effect on future orientation in high-income countries compared to upper-middle-income and lower-middle-income countries. Mothers are expected to play a more supportive and autonomy-promoting role in relation to adolescents’ future orientation, whereas fathers are expected to play a more directive and compliance-focused role (Hypothesis 6). The associations between mothers’ and fathers’ parenting and adolescents’ future orientation are expected to be moderated by cultural context, such that these associations are stronger in contexts where the corresponding value—autonomy or conformity—is more strongly endorsed.

## Method

### Participants

Participants were recruited as part of the *Parenting Across Cultures* (PAC) project, a longitudinal study examining parenting and child development across different cultural contexts. The PAC project initially included 1,338 children (50% girls; age 8, on average, at the time of recruitment), their mothers, and their fathers. The project is still ongoing, and participants have been followed annually since 2008 (more information is available at https://parentingacrosscultures.org and in Lansford et al., 2021). 82% of the parents lived together and 97% were biological parents. In the overall PAC project, families were recruited from 13 cultural groups in nine countries, including Jinan (*n* = 120) and Shanghai, China (*n* = 123); Medellín, Colombia (*n* = 108); Naples (*n* = 102) and Rome (*n* = 111), Italy; Zarqa, Jordan (*n* = 114); Kisumu, Kenya (*n* = 100); Manila, Philippines (*n* = 120); Trollhättan/Vänersborg, Sweden (*n* = 129); Chiang Mai, Thailand (*n* = 120); and Durham, NC, United States (*n* = 102 Black, *n* = 110 white, *n* = 99 Latino). The nine countries included in the study span a wide range of individualism scores according to Hofstede Insights (2023), representing some of the least to most individualistic societies globally: Colombia (score = 13), China and Thailand (score = 20), Kenya (score = 25), Jordan (score = 30), the Philippines (score = 32), Sweden (score = 71), Italy (score = 76), and the United States (score = 91). This variation allows us to explore whether parents’ self-reported individualism and collectivism values are associated with parenting practices and child adjustment across culturally diverse contexts. The countries also differ in conformity-related values, as reflected in their positions on the looseness–tightness continuum. Loose cultures—such as those with weaker social norms and greater tolerance for deviance—contrast with tight cultures, where strong norms and low tolerance for deviance prevail (Gelfand et al., 2011), suggesting broad cross-national differences in values related to conformity. In the current sample, countries ranged from among the tightest in the world (Jordan, with a score of 4.2) to among the loosest in the world (the United States, with a score of 67.9; Uz., 2015).

Participants were recruited through letters sent home by schools. Parents interested in the study either responded to the school, granting permission for researchers to contact them, or, depending on the site, schools facilitated direct contact. To ensure economic diversity, the sample included students from both private and public schools as well as families across the socioeconomic spectrum in proportions generally representative of each recruitment area. For instance, in Colombia, where socioeconomic status is categorized into six well-defined strata, families were sampled from each stratum in proportion to their representation in Medellín.

The current study focused on four time points of data collection (when participants were 10, 14, 17, and 20 years old). Participants from Sweden and China were excluded because they did not report on future orientation at the first and last time points, respectively. Consequently, the final sample consisted of 1,086 adolescents, with mean ages of 10 years at the first time point, 14 at the second, 17 at the third, and 20 at the fourth; adolescents’ mothers and fathers also participated. Mothers had an average of 12.97 years of education (*SD* = 4.03), and fathers had an average of 13.14 years of education (*SD* = 4.09).

Following the World Bank Group’s (2025) income level classification, participants’ countries were categorized into three groups: (1) high-income (*n* = 524), including Italy and the United States; (2) upper-middle-income (*n* = 228), comprising Colombia and Thailand; (3) lower-middle-income (*n* = 334), including Jordan, Kenya, and the Philippines. This income-based categorization also reflects cultural differences, with upper-middle- and lower-middle-income countries generally characterized by collectivistic cultures, whereas high-income countries tend to be more individualistic (Hofstede, 2001).

### Procedures

The measures were administered in Spanish (Colombia and the USA), Italian (Italy), Arabic (Jordan), Dholuo (Kenya), Filipino (the Philippines), Thai (Thailand), and American English (the USA and the Philippines), following a rigorous process of forward and back translation, along with methodological validation, to ensure that the instruments maintained conceptual equivalence (Erkut, 2010). Translators were fluent in both English and the target language. In addition to translating the measures, translators flagged items that were difficult to translate, culturally inappropriate, ambiguous, or elicited multiple interpretations, and provided suggestions for improvement. Country coordinators and translators reviewed these problematic items and made necessary adjustments during cross-site meetings, where all investigators and consultants discussed ambiguities and challenges on an item-by-item basis. These meetings, along with ongoing e-mail communication, ensured consistency in data collection methods across all sites. These efforts were crucial to ensure the measures were valid across all sites, ensuring linguistic equivalence and the cultural significance of all instruments (Peña, 2007). Data collection procedures were standardized across all sites, with site coordinators adhering to the same protocol for translating, back-translating, and culturally adapting the measures, conducting family interviews, and handling data entry and cleaning. Each assessment lasted between 1.5 and 2 h and was conducted at locations chosen by the participants (e.g., home, school, public library), following parent and child consent. Families were compensated with small monetary payments or in other ways (e.g., bookstore vouchers, movie tickets) endorsed by local IRBs, which approved all study procedures. The amounts and types of compensation varied across sites to be appropriately motivating without being coercive.

### Measures

#### Future orientation

Across all four waves of data collection (i.e., study Times 1, 2, 3, 4; ages 10, 14, 17, and 20), adolescents reported on their future orientation using 15 items from the Future Orientation Scale (see Steinberg et al., 2009, for details on psychometric properties and the complete scale). To minimize socially desirable responses, the measures followed a formatting approach originally developed by Harter (1982). Each question was presented in two steps. First, adolescents selected the statement that best described them from a pair of opposing statements separated by “BUT” (e.g., “*Some people like to plan things out one step at a time*,* BUT other people like to jump right into things without planning them out beforehand*”). Then, they indicated whether the chosen statement was “sort of true” or “really true” for them. This procedure created a 4-point scale ranging from “really true” for one statement to “really true” for the opposite statement. The present study focused on the overall future orientation scale, which is calculated as the mean of all 15 items. McDonald’s ω_t_ was 0.63 at age 10, 0.78 at age 14, 0.78 at age 17, and 0.76 at age 20.

#### Parental monitoring

At study Time 1 (i.e., adolescent’s age 10), mothers and fathers reported on their parental monitoring using a 10-item questionnaire based on measures from Conger et al. (1994) and Steinberg et al. (1992). Five items evaluated the extent to which parents attempt to learn about their child’s activities (e.g., *“How much do you try to know how your child spends his/her free time?”;* 0 = *do not try*, 1 = *try a little*, 2 = *try a lot*). The other five items measured how often parents set limits on their child’s activities (e.g., *“How often do you set rules or limits on who your child spends time with?”;* 0 = *never* to 3 = *always*). The standardized scores were averaged to create an overall parental monitoring composite. McDonald’s ω_t_ was 0.87 for fathers and 0.86 for mothers. For further details on this measure within the current sample, see Skinner et al. (2014).

#### Family obligations

At study Time 1 (i.e., adolescents’ age 10), mothers and fathers reported on family obligations by completing a measure developed by Fuligni et al. (1999). This instrument consists of seven items that assess the perceived significance of respecting the authority of family elders, including parents, grandparents, and older siblings (e.g., “*Please rate how important it is to you that your child treat you with great respect*”; from 1 = *not important* to 5 = *very important*). Additionally, 11 items evaluate parental expectations regarding the frequency with which children should assist with household tasks and engage in family activities (e.g., “*Please rate how often your child is expected to help out around the house*”; rated from 1 = *almost never* to 5 = *almost always*). The scores from all 18 items were averaged to generate a composite of expectations of family obligations score for each parent. McDonald’s ω_t_ was 0.87 for fathers and 0.86 for mothers.

#### Individualism and collectivism

At study Time 1 (i.e., adolescents’ age 10), mothers and fathers completed a measure of individualism and collectivism adapted from Singelis et al. (1995), Tam et al. (2003), and Triandis (1995). Parents rated the importance of different values related to autonomy and belonging to a social group and were asked whether they 1 = *strongly disagree*, 2 = *disagree*, 3 = *agree*, or 4 = *strongly agree* with each of 16 statements, 8 reflecting individualism (e.g., “I’d rather depend on myself than others”) and 8 reflecting collectivism (e.g., “To me, pleasure is spending time with others”). Items were averaged to create an individualism scale score (McDonald’s ω_t_ = 0.67 and 0.66 for fathers and mothers, respectively) and a collectivism scale score (McDonald’s ω_t_ = 0.69 and 0.65 for fathers and mothers, respectively).

#### Parent conformity values

At study Time 1 (i.e., adolescents’ age 10), mothers and fathers reported on conformity values by rating an item developed by Schwartz et al. (2001): “*I believe that people should do what they’re told. I think people should always follow rules*,* even when no one is watching*.” Parents provided their responses on a 6-point scale, ranging from 1 = *not like me at all* to 6 = *very much like me.*

#### Gender

 This variable was included in analyses as a control variable in the models.

#### Impact of the COVID-19 pandemic

Specifically, between 2021 and 2022, adolescents’ fathers and mothers completed the COVID-19 Exposure and Family Impact Survey (CEFIS; Kazak et al., 2021), which conceptualizes exposure to potentially traumatic aspects of COVID-19 and assesses the impact of the pandemic on the family. The current study used the *Impact* subscale to control for pandemic effects on future orientation when adolescents were 20 years old. The Impact scale consists of 12 items rating the pandemic’s impact on participants’ and families’ lives, as well as distress caused by the pandemic (e.g., “*In general*,* how has the COVID-19 pandemic affected each of the following: parenting?”*). McDonald’s ω_t_ was 0.66 for fathers and 0.73 for mothers.

### Analytic Plan

Preliminary analyses were carried out using IBM SPSS software 29.0.1.0; main analyses were performed using R software (version 4.2.2, Core Team, 2020) and package lavaan (Rosseel, 2012). First, Little’s (1988) Missing Completely at Random (MCAR) test was performed to compare participants with and without missing data. Second, to analyze changes in future orientation over time across multiple groups, as well as the effects of covariates, Latent Growth Curve Analysis (LGCA) was used, where latent factors represent the initial levels of statistical variables (i.e., intercepts) and their rates of change or developmental trends (e.g., slope; Burant, 2016; Byrne & Crombie, 2003; Muthén, 2002; Preacher, 2018). In LGCA, the intercept is typically a latent factor representing the initial status (i.e., the expected value at the starting point) of the trajectory across time; it is a constant for any individual across time, but it may vary between individuals. The slope is specified as a latent variable with loading reflecting time, therefore representing the rate of change across repeated measures (for more details, see Duncan & Duncan, 2009). Given the non-normal distribution of the variables, robust maximum likelihood estimation procedures were used to account for skewness or kurtosis in study variables.

Hypotheses were tested using an iterative series of multigroup latent growth curve models. First, a series of unconditional growth models with no predictors was used to examine how future orientation changes over time. Four different functional growth forms for the variables of interest in the growth models were compared. The first form was an intercept-only model where future orientation was allowed to vary in the first wave of data collection but not over time (therefore not including slope). The second form was a linear model where which assumed a constant change in future orientation over time (therefore including intercept and linear slope). The third form was a quadratic model, estimating the acceleration or deceleration of future orientation over time. The fourth form was a cubic model, estimating changes in the acceleration or deceleration of future orientation over time. Following convention (e.g., Curran & Bauer, 2011), models were compared using Satorra-Bentler chi-square likelihood ratio tests and retained the best-fitting model. A significant chi-square difference was taken as evidence that the more complex model fit the data better, whereas the more parsimonious model was retained in case of a non-significant chi-square difference. Given the limited time points of data collection, the cubic term led to estimation issues, and the best-fitting models had intercept, linear, and quadratic slopes (the results are shown in the supplemental materials). The final model was evaluated according to the following indices: the chi-square (χ^2^) statistic, the root-mean-squared error of approximation (RMSEA), the comparative fit index (CFI), and the standardized root mean squared residual (SRMR). Evaluation of model fit was based on recommended fit index cut-off values that indicate acceptable or good model fit, such as CFI > 0.95, GFI > 0.95, RMSEA < 0.08, and SRMR < 0.08 (Kline, 2023).

Once the best-fitting model was selected, predictors were added to test whether parental monitoring, family obligations, and conformity values were associated with differences in adolescents’ future orientation trajectories. Given that these variables were assessed from both mothers and fathers, it was possible to disaggregate for mothers and fathers, thus testing the effects of mothers’ and fathers’ parental monitoring, family obligations, individualism, collectivism, and conformity values on adolescents’ future orientation trajectories. Because the last wave of future orientation data collection took place in 2021–2022, after the COVID-19 pandemic, the specific effect of the COVID-19 pandemic on future orientation, collected when participants were aged 20, was also controlled. Given that no specific hypotheses were made about adolescents’ gender and future orientation, this variable was included in analyses as a control variable in the main models. Finally, two sensitivity analyses were done. First, multigroup model comparisons using the chi-square likelihood ratio were performed to examine whether future orientation developmental trajectories and predictors’ roles in these trajectories significantly differed across countries. Second, gender differences were examined to explore gender’s role and determine whether future orientation developmental trajectories and predictors’ roles in these trajectories significantly differed by gender. This study’s design and its analysis were not pre-registered. The data that support the findings of this study are available from the corresponding author upon reasonable request.

## Results

### Measurement Invariance

Measurement invariance was tested for all measures included in the current study. The alignment method (Muthén & Asparouhov, 2014) was used to test for measurement invariance in factor loadings and intercepts across cultural groups and countries with different income levels. For the future orientation measure, invariance was also tested by child gender. Across all measures, the proportion of non-invariant parameters fell below the 25% threshold suggested by Muthén and Asparouhov (2014) for acceptable non-invariance, which indicates reasonably low levels of non-invariance across cultures and countries of different income levels in the sample.

For future orientation, non-invariance was 4.8% for factor loadings and 1.9% for intercepts across cultural groups, 0% for factor loadings and 2.2% for intercepts across income countries, and 0% for factor loadings and intercepts across child gender. For parental monitoring, the alignment method demonstrated acceptable non-invariance, below 25%, in factor loadings and intercepts across cultural groups, 3.6% and 2.9% respectively, and across income countries, both 0%. For family obligations, acceptable non-invariance was observed in factor loadings and intercepts across cultural groups, 4.8% and 9.9%, and across income countries, 3.7% and 2.8%. For individualism and collectivism, factor loadings and intercepts showed non-invariance below 25% across cultural groups, 0% and 5.8%, and across income countries, 0% and 1%. Finally, for COVID-19 impact, the alignment method demonstrated acceptable non-invariance below 25% in factor loadings and intercepts across cultural groups, 6.3% and 14.3%, and across income countries, 0% and 7.4%.

### Descriptive Analyses

Tables 1 and 2 report descriptive analyses and bivariate correlations between variables. The MCAR test was significant, χ^2^ (477) = 801.866, *p* < .001. However, the normed χ2/df of 1.6810 suggests that data were missing at random, supporting the inclusion of participants with missing data in the analyses and the use of full information maximum likelihood to handle missing data (Bollen, 1989).

**Table 1:** Descriptive Statistics for Future Orientation and Parenting Dimensions by Parent Gender Across Countries

	Future Orientation				Parental Monitoring		Family Obligations		Individualism		Collectivism		Conformity Values	
	Age 10 (M, SD)	Age 14 (M, SD)	Age 17 (M, SD)	Age 20 (M, SD)	Mothers (M, SD)	Fathers (M, SD)	Mothers (M, SD)	Fathers (M, SD)	Mothers (M, SD)	Fathers (M, SD)	Mothers (M, SD)	Fathers (M, SD)	Mothers (M, SD)	Fathers (M, SD)
Colombia (*N* = 108)	2.95 (0.38)	2.85 (0.46)	2.89 (0.46)	2.91 (0.37)	0.26 (0.52)	0.22 (0.59)	4.29 (0.36)	4.35 (0.44)	2.67 (0.36)	2.66 (0.34)	3.43 (0.29)	3.28 (0.33)	2.50 (1.53)	2.17 (1.11)
Italy (*N* = 213)	2.72 (0.41)	2.66 (0.44)	2.84 (0.46)	2.90 (0.43)	0.18 (0.48)	− 0.09 (0.63)	3.96 (0.45)	3.85 (0.49)	2.65 (0.39)	2.72 (0.41)	3.22 (0.31)	3.22 (0.30)	2.99 (1.32)	2.75 (1.19)
Kenya (*N* = 100)	2.89 (0.43)	3.33 (0.46)	3.42 (0.50)	3.18 (0.50)	− 0.26 (0.59)	− 0.01 (0.71)	3.66 (0.56)	3.69 (0.54)	2.69 (0.47)	2.82 (0.52)	3.18 (0.42)	3.18 (0.44)	2.48 (1.10)	2.45 (1.09)
Jordan (*N* = 114)	2.91 (0.49)	2.77 (0.63)	2.84 (0.49)	2.89 (0.45)	0.23 (0.41)	0.09 (0.68)	4.23 (0.39)	4.18 (0.46)	3.10 (0.37)	3.15 (3.24)	3.24 (0.34)	3.24 (0.39)	2.09 (1.12)	2.17 (1.09)
Philippines (*N* = 120)	3.00 (0.39)	3.00 (0.45)	3.08 (0.49)	2.95 (0.43)	0.04 (0.63)	0.12 (0.55)	4.10 (0.51)	3.95 (0.50)	2.9 (0.42)	2.9 (0.41)	3.42 (0.33)	3.34 (0.33)	2.28 (1.18)	2.32 (1.13)
Thailand (*N* = 120)	2.91 (0.43)	2.97 (0.52)	3.02 (0.51)	2.79 (0.40)	− 0.27 (0.62)	− 0.00 (0.65)	4.00 (0.42)	3.98 (0.48)	2.61 (0.35)	2.69 (0.35)	3.30 (0.32)	3.31 (0.36)	3.34 (1.15)	3.28 (1.15)
United States (*N* = 311)	2.91 (0.45)	2.93 (0.52)	3.04 (0.49)	3.04 (0.46)	0.26 (0.53)	0.23 (0.60)	3.78 (0.52)	3.78 (0.53)	2.63 (0.45)	2.69 (0.38)	3.35 (0.34)	3.32 (0.34)	2.42 (1.27)	2.54 (1.28)

Note. M = Mean. SD = Standard Deviation

**Table 2:** Bivariate correlations among future orientation and parenting dimensions across adolescence

	(1)	(2)	(3)	(4)	(5)	(6)	(7)	(8)	(9)	(10)	(11)	(12)	(13)	(14)
(1) Future Orientation Age 10	1.00													
(2) Future Orientation Age 14	0.31**	1.00												
(3) Future Orientation Age 17	0.23**	0.47**	1.00											
(4) Future Orientation Age 20	0.13**	0.35**	0.49**	1.00										
(5) Parental Monitoring (mothers)	− 0.00	− 0.05	− 0.07	− 0.01	1.00									
(6) Parental Monitoring (fathers)	0.08*	0.03	0.01	0.04	0.19**	1.00								
(7) Family Obligations (mothers)	0.07*	− 0.04	− 0.06	− 0.01	0.29**	0.17**	1.00							
(8) Family Obligations (fathers)	0.08*	− 0.07	− 0.03	− 0.03	0.18**	0.33**	0.44**	1.00						
(9) Individualism (mothers)	0.07*	− 0.03	− 0.03	− 0.08*	0.14**	0.08*	0.25**	0.12**	1.00					
(10) Individualism (fathers)	0.01	0.02	0.00	0.02	0.01	0.09*	0.12**	0.18**	0.27**	1.00				
(11) Collectivism (mothers)	0.09*	− 0.00	− 0.01	− 0.02	0.23**	0.17**	0.32**	0.15**	0.27**	0.02	1.00			
(12) Collectivism (fathers)	0.13**	0.01	0.05	− 0.02	0.09*	0.33*	0.17**	0.36**	0.10*	0.22**	0.26**	1.00		
(13) Conformity Values (mothers)	0.04	− 0.02	− 0.07*	− 0.05	− 0.16**	− 0.02	− 0.15**	− 0.03	− 0.13**	− 0.08*	− 0.19**	− 0.09*	1.00	
(14) Conformity Values (fathers)	− 0.02	0.00	0.01	− 0.05	− 0.08	− 0.19**	− 0.14**	− 0.23**	− 0.08*	− 0.17**	− 0.14**	− 0.18**	0.22**	1.00

*p < .05. **p < .001

### Future Orientation Trajectories across Adolescence

First, how future orientation changes across adolescence was examined. Table 3 reports the results of the unconditional (i.e., without predictors) models. At age 10 (time 1), individuals in high- and upper-middle-income countries score an average of 7.59, variance = 0.14, and 11.71, variance = 0.06, respectively, on the future orientation scale. However, neither the linear nor the quadratic slopes are significant, suggesting that, on average, future orientation does not exhibit a significant and consistent change over time. Conversely, at age 10 (time 1), individuals in the lower-middle-income countries score an average of 28.94, variance = 0.01, on the future orientation scale. This score significantly increases over time, at a constant rate of 0.55 points every time point. Still, the negative and significant mean of the quadratic slope indicates that this growth is slow and eventually declines.

**Table 3:** Unconditional multigroup latent growth curve model results by country income level

	β	SE	Variance
**High Income Countries**			
Intercept	**7.59**	0.04	**0.014**
Linear slope	0.11	0.03	**0.014**
Quadratic slope	0.05	0.01	0.01
**Upper-Middle Income Countries**			
Intercept	**11.71**	0.03	0.06
Linear slope	0.18	0.05	0.07
Quadratic slope	− 0.31	0.02	0.01
**Lower-Middle Income Countries**			
Intercept	**28.94**	0.06	0.01
Linear slope	**0.55**	0.04	0.10
Quadratic slope	**− 0.46**	0.01	0.01
**Fit indices**			
χ^2^ (df)	**17.284 (3)**		
RMSEA	**0.120**		
CFI	0.969		
GFI	1.00		
SMRM	0.027		

Note. Standardized estimates are reported, and bold values refer to *p* < .05; SE = standard error; RMSEA = root mean square error of approximation; CFI = comparative fit index; GFI = goodness of fit index; SRMR = standardized root mean square residual

### Predictors of Future Orientation Trajectories across Adolescence

Next, the roles of parenting and other factors in adolescents’ future orientation were explored. Table 4 reports the results of the conditional (i.e., with predictors) models. Maternal and paternal monitoring are significantly associated with future orientation intercept (i.e., future orientation’s level at study time 1), β= − 0.38, SE = 0.06, *p* = .044; β = 0.47, SE = 0.05, *p* = .009, respectively, only in lower-middle-income countries, meaning that high paternal monitoring is associated with a higher initial level of future orientation during pre-adolescence while high maternal monitoring is related to a lower initial level of future orientation. No associations between parents’ family obligation expectations and adolescents’ future orientation emerge in high-income countries, whereas both maternal and paternal family obligation expectations are significantly associated with adolescents’ future orientation in upper-middle-income and lower-middle-income countries. Specifically, mothers’ and fathers’ family obligation expectations are related to adolescents’ future orientation intercept (i.e., future orientation’s level at study time 1), β = 0.39, SE = 0.08, *p* = .013; β = − 0.45, SE = 0.07, *p* = .012, respectively, in upper-middle-income countries. In these countries, higher maternal family obligation expectations are related to higher future orientation at age 10, whereas higher paternal family obligation expectations are related to lower future orientation at age 10. Finally, in lower-middle-income countries, mothers’ family obligation expectations are significantly associated with future orientation’s linear, β = − 0.29, SE = 0.09, *p* = .047, and quadratic, β = 0.39, SE = 0.03, *p* = .008 slopes, suggesting that high maternal family obligation expectations are connected to a significant decline and a subsequent acceleration in the growth of future orientation over time.

**Table 4:** Conditional multigroup latent growth curve model results of parenting dimensions by parent gender across country income level

	Parental Monitoring (M)	Parental Monitoring (F)	Family Obligations (M)	Family Obligations (F)	Individualism (M)	Individualism (F)	Collectivism (M)	Collectivism (F)	Conformity Values (M)	Conformity Values (F)
	β (SE)	β (SE)	β (SE)	β (SE)	β (SE)	β (SE)	β (SE)	β (SE)	β (SE)	β (SE)
	**High Income Countries**									
Intercept	0.11 (0.04)	− 0.05 (0.04)	0.03 (0.05)	− 0.09 (0.07)	0.00 (0.06)	− 0.04 (0.07)	0.08 (0.08)	**0.28 (0.09)**	0.09 (0.02)	0.09 (0.02)
Linear slope	− 0.10 (0.07)	0.02 (0.07)	− 0.05 (0.08)	− 0.16 (0.09)	− 0.06 (0.08)	0.06 (0.11)	0.07 (0.11)	0.02 (0.13)	**− 0.19 (0.03)**	0.02 (0.03)
Quadratic slope	0.12 (0.02)	0.02 (0.02)	0.04 (0.03)	0.27 (0.03)	0.08 (0.03)	− 0.06 (0.03)	0.11 (0.03)	− 0.19 (0.04)	0.18 (0.01)	− 0.06 (0.01)
	**Upper-Middle Income Countries**									
Intercept	0.11 (0.05)	− 0.05 (0.05)	**0.39 (0.08)**	**− 0.45 (0.07)**	− 0.02 (0.09)	0.02 (0.09)	0.21 (0.09)	− 0.17 (0.09)	0.19 (0.02)	− 0.02 (0.02)
Linear slope	0.15 (0.09)	0.04 (0.07)	− 0.46 (0.13)	− 0.32 (0.11)	− 0.16 (0.14)	0.31 (0.14)	− 0.21 (0.16)	0.20 (0.16)	0.17 (0.04)	− 0.02 (0.04)
Quadratic slope	− 0.18 (0.03)	0.06 (0.03)	0.38 (0.04)	0.46 (0.04)	0.07 (0.04)	− 0.31 (0.04)	0.15 (0.05)	− 0.23 (0.05)	− 0.21 (0.01)	− 0.01 (0.01)
	**Lower-Middle Income Countries**									
Intercept	**− 0.38 (0.06)**	**0.47 (0.05)**	− 0.04 (0.07)	0.17 (0.06)	0.10 (0.07)	− 0.11 (0.06)	0.12 (0.07)	0.14 (0.07)	0.24 (0.03)	− 0.02 (0.03)
Linear slope	0.13 (0.08)	0.07 (0.08)	**− 0.29 (0.09)**	− 0.12 (0.10)	**− 0.27 (0.10)**	0.02 (0.10)	− 0.12 (0.13)	− 0.02 (0.13)	**− 0.32 (0.04)**	0.13 (0.05)
Quadratic slope	− 0.12 (0.03)	− 0.13 (0.03)	**0.39 (0.03)**	0.01 (0.03)	0.16 (0.03)	0.04 (0.03)	0.08 (0.04)	0.03 (0.04)	**0.31 (0.01)**	− 0.15 (0.02)

Note. Values represent standardized regression coefficients (*β*) with standard errors (SE) in parentheses. Bold values refer to *p* < .05. M = mother; F = father

Mothers’ individualism and fathers’ collectivism are significantly associated with future orientation’s linear slope, β = − 0.27, SE = 0.10, *p =* .05, in lower-middle-income countries and intercept (i.e., future orientation’s level at study time 1), β = 0.28, SE = 0.09, *p* < .001. Therefore, in lower-middle-income countries, high maternal individualism is related to a continuous decline of future orientation over time, whereas in high-income countries, high paternal collectivism is associated with a higher future orientation at age 10.

No significant associations between fathers’ conformity values and adolescents’ future orientation emerge. Conversely, maternal conformity values are significantly connected with future orientation linear slope in high-income countries, β = − 0.19, SE = 0.03, *p* = .019, and lower-middle-income countries, β =-0.32, SE = 0.04, *p* = .028. Maternal conformity values are also associated with future orientation quadratic slope in lower-middle-income countries, β = 0.31, SE = 0.01, *p* = .026. Specifically, high maternal conformity values in both high-income and lower-middle-income countries are related to a continuous decline of future orientation over time. Only in lower-middle-income countries, they are also significantly linked to a subsequent increase in future orientation over time.

Finally, although Table 4 does not report COVID-19 effects on future orientation, all models include this control variable. No effects of COVID-19 emerge in any country. The final model fit all data well, χ2 (54) = 52.115, *p* = .057, RMSEA = 0.00, CFI = 1.00, GFI = 0.999, SRMR = 0.017, and the predictors explain a percentage of variance varying by group, R^2^_high−income countries intercept_ = 0.10, R^2^_upper−middle−income countries intercept_ = 0.34, R^2^_lower−middle−income countries intercept_ = 0.53, R^2^_high−income countries linear slope_= 0.10, R^2^_upper−middle−income countries linear slope_ = 0.86, R^2^_lower−middle−income countries linear slope_ = 0.31, R^2^_high−income countries quadratic slope_= 0.16, R^2^_upper−middle−income countries quadratic slope_= 0.89, R^2^_lower−middle−income countries quadratic slope_= 0.25.

### Sensitivity Analyses

#### Multigroup model comparisons

To identify exactly which trajectory and regression paths differed across countries, multigroup model comparisons were performed using the chi-square likelihood ratio test (see Supplemental Table 2 for the results). First, intercepts, slopes, and regression paths were constrained to be equal across the three income groups (i.e., high-income, upper-middle-income, and lower-middle-income countries). Then, one by one, these paths were freed to vary across groups, and the differences in fit were compared using a 1-degree-of-freedom chi-square test. If the chi-square test revealed that the model fits better when the path is allowed to vary freely across groups, the path remains unconstrained. Otherwise, it remained equal across groups. After examining chi-squared difference tests, the model predicting future orientation trajectories fit best when the intercept, linear, and quadratic slopes, and regression paths were free to vary between all countries. This indicated that the grouping division is really accounting for heterogeneities across different countries.

#### Gender differences in future orientation trajectories and their predictors

To test for significant gender differences in future orientation patterns over time and across countries, the original sample was divided into females and males, and separate multigroup latent growth curve models were run by gender. To examine the changes in future orientation across time for females and males, (1) intercept-only model, (2) intercept and linear slope model, (3) intercept, linear, and quadratic slope model, and (4) intercept, linear, quadratic, and cubic slope model were compared using Satorra-Bentler chi-squared likelihood ratio tests. For both females and males, both quadratic and cubic terms led to estimation issues, and the best-fitting model included the intercept and the linear slope. Once the best-fitting model was selected, predictors of future orientation (i.e., parental monitoring, family obligations, individualism vs. collectivism, and conformity values) were added. Finally, to identify precisely which trajectory and regression paths differed across countries for females and males, multigroup model comparisons were performed using the chi-square likelihood ratio test (using the same procedure described above). For females, the model predicting future orientation trajectories fit best when the intercept, the linear slope, and the regression paths were free to vary between all countries. For males, the model predicting future orientation trajectories fit best when the intercept and the linear slope were free to vary, whereas the regression paths were constrained to be equal across countries. These results highlight that, for females, there are significant country differences in how future orientation changes over time and is influenced by parental monitoring, family obligations, and conformity values. On the contrary, there are significant country differences for males in how future orientation changes over time, but not in how parental monitoring, family obligations, individualism vs. collectivism, and conformity values influence future orientation. Supplemental Tables 4 and 5 report the results (for clarity, only the final model results are reported in the supplemental material, but model comparison information is available by emailing the corresponding author).

The results show that, only for females in lower-middle-income countries, higher maternal and paternal monitoring are significantly associated with lower and higher future orientation at study time 1, respectively. No effects of parental monitoring on future orientation emerge for males. Moreover, only for females, paternal high family obligation expectations are related to lower future orientation at study time 1 in high- and upper-middle-income countries, to a constant increase of future orientation in upper-middle-income countries, and a constant decrease in lower-middle-income countries. No effects of family obligation expectations on future orientation emerge for males. As for parents’ individualism and collectivism, mothers’ high individualism is associated with a constant decrease in future orientation over time (only for females in upper-middle-income countries). Mothers’ high collectivism is related to a higher future orientation in study year 1 (only for females in high-income countries) and a constant decrease in future orientation over time (only for females in upper-middle-income countries). No effects of maternal individualism and collectivism on future orientation emerge for males. On the contrary, for both females and males, fathers’ high collectivism is associated with higher future orientation in study year 1 and a constant decrease of future orientation over time. This association is significant for females living in high-income countries and lower-middle-income countries, whereas no significant country differences in how parents’ cultural values influence future orientation emerge for males. Finally, maternal high family obligation expectations are associated with a constant decrease in future orientation over time (only for females in high-income countries) and a higher future orientation at study time 1 (only for females in upper-middle-income countries). Similarly, in the male sample, paternal high family obligation expectations are associated with a higher future orientation at study time 1 and a constant decrease in future orientation over time. However, no significant country differences in how parents’ conformity values influence future orientation are found for males.

## Discussion

To date, there is limited understanding of how parenting dimensions influence adolescents’ future orientation over time across different socioeconomic and cultural contexts. To fill this gap, the present study investigates changes in adolescents’ future orientation and how dimensions of mothers’ and fathers’ parenting —parental monitoring, family obligations, individualism vs. collectivism, and conformity values—are associated with adolescents’ future orientation across countries that differ in income level (high, upper-middle, and lower-middle) and cultural contexts. Notably, this study examines these factors using a longitudinal and cross-cultural approach. Overall, the findings suggest that parenting dimensions, particularly family obligations and conformity values, play a meaningful role in shaping adolescents’ future orientation across diverse contexts.

Contrary to expectations (Hypothesis 1, Hypothesis 2), individuals living in lower-middle-income countries exhibited the highest levels of future orientation at age 10, followed by a significant increase during adolescence, which eventually slowed and declined. In contrast, adolescents from high-income countries exhibit the lowest initial levels of future orientation, followed by those from upper-middle-income countries; in both groups, future orientation remains stable through adolescence. Although these findings challenge the predictions of life history theory which suggests that resource abundance and higher economic status foster future orientation, they are consistent with other studies (Park et al., 2024). The findings suggest that individuals in high-income countries, where resources are abundant, may perceive less pressure to plan for the future, whereas people in resource-scarce environments may be more motivated to think ahead, as limited opportunities create a greater sense of urgency (Mullainathan & Shafir, 2013). In addition, in lower-middle-income countries, young people may initially develop a strong future orientation, driven by greater access to education, family responsibilities, and aspirations for upward mobility. However, a subsequent slowdown or decline in this orientation may reflect growing awareness of structural barriers and economic instability, which undermine the feasibility of long-term planning and dampen motivation to invest in the future (Chen & Guo, 2024).

Regarding parental monitoring, a negative association with the development of future orientation was hypothesized (Hypothesis 3), and partial support for this hypothesis was found. In lower-middle-income countries, higher maternal monitoring in pre-adolescence is linked to lower levels of future orientation, suggesting that excessive control may hinder adolescents’ autonomy and future planning—particularly in constrained socio-economic contexts (Liu et al., 2024). Conversely, contrary to expectations, higher paternal monitoring is associated with greater initial future orientation. This may reflect adolescents’ perception of paternal monitoring as more supportive and structuring rather than controlling, especially in lower-middle-income settings (Lin, 2023). Notably, neither maternal nor paternal monitoring is related to changes over time, indicating that early monitoring shapes initial levels of future orientation but not its developmental trajectory. No significant associations between parental monitoring and future orientation emerge in high- and upper-middle-income countries, perhaps reflecting the presence of stronger institutional support and cultural expectations that promote adolescent autonomy, thereby reducing the influence of parental practices.

A complex picture regarding family obligation expectations in upper-middle-income countries emerges (Hypothesis 4), as maternal and paternal influences show opposite patterns: maternal expectations are positively linked to adolescents’ future orientation at age 10, whereas paternal expectations are negatively associated with adolescents’ future orientation. These differences may reflect distinct socialization roles in the family. Mothers, who the literature points out are often characterized by more nurturing and responsive parenting (Yaffe, 2023), may encourage the internalization of family obligations in ways that support long-term goal setting and future planning (Wang & Tamis-LeMonda, 2003). Conversely, fathers, typically found to be more authoritarian and focused on obedience, may convey expectations perceived as rigid or imposed, potentially weakening adolescents’ intrinsic motivation and undermining future orientation. The present findings were consistent with a previous study (Knight & Sayegh, 2010) that highlights how cultural values such as obedience and authority can have ambivalent effects—at times reinforcing a sense of obligation but also creating constraining pressures that undermine well-being and motivation. This contrast may be particularly relevant in societies experiencing social and economic transition, as these societal changes may reshape traditional parenting roles and influence adolescents’ perceptions of parental authority. Conversely, in lower-middle-income countries, maternal family obligation expectations are linked to a more complex developmental trajectory—characterized by an initial decline in future orientation followed by a later increase. This nonlinear pattern partially supports the hypothesis and may reflect underlying developmental conflicts. Initially, high maternal family obligation demands may be experienced as burdensome by adolescents, particularly in contexts where material and emotional resources are limited and where such obligations often involve concrete responsibilities. These demands can diminish adolescents’ motivation to invest in long-term personal goals (Knight & Sayegh, 2010). However, as adolescents mature, they may come to reinterpret family obligations not as constraints but as meaningful sources of identity, direction, and purpose—especially in collectivistic cultures that emphasize loyalty, family cohesion, and shared responsibility (Knight & Sayegh, 2010). Consistent with the findings from the present study, a study of Taiwanese adolescents found that those endorsing collectivistic values were more likely to develop a clear and consolidated identity, indicating that family obligations can be integrated into adolescents’ sense of self rather than experienced merely as limitations (Lee et al., 2010). The results are also partially in line with another study that found that family obligation values were positively linked to supportive parenting, self-determined motivation, and well-being in Korean students, but showed weaker or no associations in European American students (Oh et al., 2020). Finally, in high-income countries, no significant associations emerge between parents’ family obligation expectations during pre-adolescence and adolescents’ future orientation. This finding suggests that, in individualistic cultures, adolescents’ future orientation may be less influenced by parental expectations regarding family obligations, compared to collectivistic cultures. Additionally, greater economic resources reduce the need for adolescents to contribute to family responsibilities, allowing them to focus more freely on individual aspirations. As a result, even when family obligation expectations exist, they may be less strongly internalized and thus less influential in shaping adolescents’ future orientation.

The results partially support Hypothesis 5 regarding the role of parents’ cultural orientations in shaping adolescents’ future orientation. Interestingly, in high-income countries—typically characterized by individualistic values—paternal collectivism is positively associated with adolescents’ initial future orientation. In this study, adolescents from high-income countries generally show lower levels of future orientation because greater economic security reduces adolescents’ urgency to plan ahead. In this context, paternal collectivist values—centered on group cohesion and shared responsibility—may serve as a compensatory influence, encouraging adolescents to adopt a longer-term perspective that goes beyond personal achievement. In lower-middle-income countries, higher maternal individualism is associated with a decline in future orientation over time, possibly reflecting the challenges adolescents face in contexts with limited resources. Here, emphasizing independence may increase stress and undermine positive future planning (Lee et al., 2010). In upper-middle-income countries, no significant associations emerge between parental cultural orientations and adolescents’ future orientation, possibly due to the coexistence or fluctuation of cultural values weakening parental influence.

Regarding parental conformity values, higher maternal conformity values are associated with a decline of future orientation over time in high-income countries and lower-middle-income countries, and linked to a subsequent increase in future orientation only in lower-middle-income countries. In high-income countries, higher maternal conformity is linked to a steady decline in future orientation over time, possibly reflecting misalignments between conformity and the individualistic values predominant in these societies, where autonomy and self-expression are highly valued (Knight & Sayegh, 2010). In contrast, in lower-middle-income countries, maternal conformity is related to a nonlinear trajectory of future orientation, pointing to a complex developmental process, where adolescents may first perceive conformity expectations as motivating or protective, then as restrictive during early/middle adolescence, before eventually integrating these values into a coherent future-oriented identity. These results align with the cultural emphasis on communal goals and shared responsibilities typical of collectivistic countries, which may facilitate adolescents’ reconciliation of conformity with their personal aspirations (Oh et al., 2020). No effects of parental conformity emerge in upper-middle-income countries, prompting the need for further research on the topic.

Regarding the differentiated roles of mothers and fathers, the findings offer partial support for Hypothesis 6, which posits that mothers would play a more supportive and autonomy-promoting role in shaping adolescents’ future orientation, whereas fathers would adopt a more directive and conformity-oriented approach. Instead, the results suggest a more complex picture with mother and father influences conditioned by context. In lower-middle-income countries, paternal monitoring is positively associated with higher initial levels of future orientation in preadolescence, indicating that fathers play a supportive and structure-providing role during early developmental stages. Conversely, maternal monitoring is linked to lower levels of future orientation, potentially reflecting perceptions of excessive control that may hinder pre-adolescents’ growing desire for autonomy. With respect to family obligation, an inverse pattern emerges in upper-middle-income countries, with maternal family obligation expectations being associated with higher levels of future orientation in pre-adolescence and paternal expectations with lower levels. In lower-middle-income countries, only maternal family obligation expectations significantly influence future orientation over time, suggesting changing developmental trajectories. In high-income countries, only paternal collectivism is positively associated with future orientation in pre-adolescence, whereas in lower-middle-income countries, maternal individualism is negatively linked to changes in future orientation over time. Regarding conformity values, only maternal conformity shows significant associations with adolescents’ future orientation, underscoring mothers’ central role in transmitting cultural and familial norms. In contrast, fathers’ values in these domains do not significantly predict future orientation in any context. Overall, these findings support the idea of differentiated parental roles (Yaffe, 2023), while also highlighting that these roles are not uniform. Instead, they vary depending on the specific parenting domain, cultural context, and stage of adolescent development—challenging overly simplified assumptions about parental influence (Gorla et al., 2024). Moreover, these results reflect the broader transformations taking place within contemporary families. Changes such as declining marriage rates, increasing divorce rates, and the diversification of family structures , ) illustrate an evolving social context in which traditional parental roles are being redefined and reshaped to meet new relational and developmental demands.

Finally, analyzing the data separately for males and females, the results indicate that gender does not substantially alter the overall trajectory of future orientation during adolescence. However, important gender differences emerge when considering specific parental and cultural dimensions. For females, higher maternal and paternal monitoring, family obligation expectations, and maternal individualism or collectivism are significantly associated with variations in future orientation, with effects varying across income groups. In contrast, males show fewer and more limited effects, with only paternal collectivism and paternal family obligation expectations influencing their future orientation, generally consistently across countries. These patterns may reflect a domain-specific gender difference in future orientation: females tend to be more oriented toward family-related future goals, whereas males are more focused on work or career-related goals (Greene & DeBacker, 2004; Seginer & Lilach, 2004). Consequently, parenting dimensions may have a stronger influence on females, whose future orientation is more closely linked to family dynamics. Overall, although the developmental trajectory of future orientation is similar for both genders, females appear more sensitive to a broader range of parenting and cultural factors.

Taken together, these findings suggest that adolescents’ future orientation emerges from an interplay of parenting practices, socio-economic context, and cultural values. Across countries, lower-middle-income adolescents show the highest initial levels that rise then slow; adolescents from upper-middle-income countries exhibit intermediate and stable levels, whereas high-income adolescents show consistently lower levels, indicating that resources alone do not determine future orientation. Parental influences vary by domain and context: maternal monitoring and conformity can either support or hinder future orientation depending on developmental stage and socio-cultural conditions, whereas paternal behaviors, including monitoring and collectivist values, often provide structure or compensatory guidance. Family obligation expectations also show subtle effects, with maternal expectations generally promoting long-term planning in collectivistic societies; paternal expectations sometimes constrain adolescents’ future orientation. All in all, the results point to family obligations and conformity values as the parenting dimensions most strongly related to adolescents’ future orientation. Looking at gender differences, the trajectory of future orientation during adolescence is similar for males and females, but females are more influenced by a range of parental and cultural factors, whereas males show more limited effects. Overall, the findings underscore the importance of considering both relational and contextual factors in analyzing future orientation, highlighting that parental roles are dynamic and embedded within socio-economic and cultural contexts.

### Strengths and Limitations

Key strengths of this study include its longitudinal and cross-cultural design, the inclusion of both maternal and paternal roles, and the analysis of developmental patterns across countries with varying income levels. The results demonstrate that future orientation follows nonlinear trajectories throughout adolescence and that parental influences on adolescents’ future orientation vary depending on the specific parenting dimension (parental monitoring, family obligations, individualism vs. collectivism, and conformity values), on mother or father, and on broader economic and cultural contexts. Overall, the study highlights the need to account for socioeconomic and cultural variability when examining developmental processes. However, this study has several limitations that should be acknowledged. First, unlike the other dimensions, conformity values in this study were assessed using a single-item measure. Although this represents a limitation in terms of construct depth, this approach has been widely adopted in previous research (Lansford, 2024) and has demonstrated acceptable validity in capturing this specific value dimension. Second, although the samples were locally representative of the sites from which they were drawn, they were not nationally representative. Therefore, the findings should not be generalized to entire countries or to cultural groups beyond those included in the study. Third, other relevant factors that might play a significant role in shaping how parenting influences the development of adolescents’ future orientation are not considered, such as maternal and paternal autonomy-supportive parenting (Seginer et al., 2004) or family socioeconomic status (Song & Sun, 2025). Although family socioeconomic status is undoubtedly important, this study aimed to capture broader socioecological influences by comparing countries at different income levels. This approach makes it possible to examine how shared economic and cultural systems—beyond individual family circumstances—are related to developmental trajectories of adolescents’ future orientation. Expanding the range of examined factors and moderators remains a valuable direction for future research to deepen understanding of how parenting dimensions contribute to adolescents’ future orientation. Then, given that future orientation data were collected at four time points, more complex forms of change (i.e., cubic slope) led to estimation issues, so linear and quadratic slopes were the focus of this work. Finally, future orientation longitudinal data after COVID-19 are not available, limiting possibilities to test for longitudinal effects of the pandemic.

### Practical Implications

The present study advances understanding of adolescence by showing how parenting practices within diverse socioeconomic and cultural contexts shape adolescents’ future orientation. The findings underscore the importance of educational policies and interventions that consider these contextual factors and the influence of family dynamics on youth development. In particular, family-focused programs that enhance parental responsiveness and support a balance between family obligations and adolescent autonomy may effectively promote the development of future orientation across varied settings.

## Conclusion

This study advances understanding of how parenting practices and family dynamics shape future orientation across diverse socioeconomic and cultural contexts by providing longitudinal data from seven countries with different income levels and cultural orientations. The findings indicate that future orientation emerges from the interplay of parenting practices, socioeconomic conditions, and cultural values, with developmental trajectories varying across income groups. In particular, family obligations and conformity values were the parenting dimensions most strongly related to adolescents’ future orientation. Males and females showed similar developmental trajectories, although females were somewhat more influenced by parental and cultural factors. These results highlight the importance of specific parenting dimensions in shaping adolescents’ future orientation across diverse contexts.

## Supplementary Information

Below is the link to the electronic supplementary material.

Supplementary Material 1

## Data Availability

The datasets analyzed during the current study are not publicly available but are available from the corresponding author on reasonable request.
