# Supplementary material for: Future Orientation in Adolescents: Development and the Roles of Parenting in Different Income Countries
Source: J Youth Adolesc. 2025 Nov 25;55(4):964–81. doi: 10.1007/s10964-025-02288-4 (PMC12961908; doi:10.1007/s10964-025-02288-4)
Supplement: Supplementary file 1 — Supplementary Material 1 [file 10964_2025_2288_MOESM1_ESM.docx]

**Supplemental Table 1**

*Unconditional Model Comparisons using Chi-square Likelihood Ratio Tests Examining Future Orientation Across Country Income Levels*

|  | First model | | | Second model | | | Third model | | |
| --- | --- | --- | --- | --- | --- | --- | --- | --- | --- |
|  | β (SE) | | | β (SE) | | | β (SE) | | |
|  | High Income Countries | Upper-Middle  Income Countries | Lower-  Middle  Income Countries | High Income Countries | Upper-Middle  Income Countries | Lower-  Middle  Income Countries | High Income Countries | Upper-Middle  Income Countries | Lower-  Middle  Income Countries |
| Intercept | **9.95 (.02)** | **12.87 (.02)** | **10.86 (.02)** | **8.65 (.02)** | **11.69 (.03)** | **11.25 (.03)** | **7.59 (.02)** | **11.71 (.03)** | **28.95 (.03)** |
| Linear slope | - | - | - | **.39 (.01)** | **-.32 (.01)** | **.17 (.01)** | .11 (.03) | .18 (.05) | **.55 (.04)** |
| Quadratic slope | - | - | - | - | - | - | .05 (.01) | -.31 (.02) | **-.46 (.01)** |
| Chi-square likelihood ratio tests | | | | | | | | | |
| AIC | 4099.9 | | | 4002.1 | | | 3970.1 | | |
| BIC | 4188.0 | | | 4134.4 | | | 4161.2 | | |
| P (> Chisq) |  | | | **<.001** | | | **<.001** | | |

*Note.* The table reports the iterative series of multigroup latent growth curve models performed to examine how future orientation changes over time. The first model refers to an intercept-only model where future orientation was allowed to vary in the first wave of data collection but not over time (therefore not including slope). The second model refers to a linear model where we assumed a constant change in future orientation over time (therefore including intercept and linear slope). The third model refers to a quadratic model, where the acceleration or deceleration over time in future orientation was estimated. A fourth form (i.e., a cubic model estimating changes in the acceleration or deceleration in future orientation over time) was also tested, but it is not reported as it led to estimation issues. In the table, standardized estimates are reported, and bold values refer to *p* < .05. The symbol – indicates that the form (i.e., slopes) was not tested in the model. Values represent unstandardized regression coefficients (*β*) with standard errors (SE) in parentheses. AIC = Akaike information criterion; BIC = Bayesian information criterion.

**Supplemental Table 2**

*Multigroup Model Comparisons using Chi-square Likelihood Ratio Tests: Sensitivity Analysis Across the Entire Sample*

|  | First model | Second model | Third model | Fourth model | Fifth model |
| --- | --- | --- | --- | --- | --- |
| χ2 (df) | **214.2881 (130)** | **192.239(128)** | **168.963 (126)** | **155.846 (124)** | **52.115 (54)** |
| RMSEA | .042 | .037 | .031 | .027 | .000 |
| CFI | .838 | .877 | .918 | .939 | 1.00 |
| GFI | .997 | .998 | .998 | .998 | .999 |
| SMRM | .043 | .038 | .036 | .035 | .017 |
| chi-square likelihood ratio tests | | | | | |
| AIC | -4526.0 | -4544.7 | -4564.0 | -4573.1 | -4536.8 |
| BIC | -4186.7 | -4195.4 | -4204.7 | -4203.8 | -3818.2 |
| P  (> Chisq) |  | **<.001** | **<.001** | **<.001** | **.005** |

*Note.* The table reports the multigroup latent growth curve model comparison performed as the first sensitivity analysis with the entire sample (not looking at gender differences). The first model refers to a model where intercept, linear, and quadratic slopes and regression paths were all constrained across countries. The second model refers to a model where the intercept is free to vary across countries, whereas linear and quadratic slopes and regression paths are constrained to be equal across countries. The third model refers to a model where both the intercept and the linear slope are free, whereas the quadratic slope and regression paths are constrained to be equal across countries. The fourth model refers to a model where the intercept, the linear slope, and the quadratic slope are free, whereas the regression paths are constrained to be equal across countries. Finally, the fifth model refers to a model where everything is free to vary across countries. In the table, standardized estimates are reported, and bold values refer to *p* < .05. χ² = chi-square test of model fit with degrees of freedom in parentheses; RMSEA = root mean square error of approximation; CFI = comparative fit index; GFI = goodness of fit index; SRMR = standardized root mean square residual; AIC = Akaike information criterion; BIC = Bayesian information criterion; *p* (> χ²) = probability associated with the chi-square test.

**Supplemental Table 3**

*Unconditional Model Comparisons using Chi-square Likelihood Ratio Tests of Future Orientation Separately for Males and Females Across Countries*

|  | First model | | | Second model | | | | |
| --- | --- | --- | --- | --- | --- | --- | --- | --- |
|  | β (SE) | | | β (SE) | | | | |
|  | High Income Countries | Upper-Middle  Income Countries | Lower-  Middle  Income Countries | High Income Countries | Upper-Middle  Income Countries | | Lower-  Middle  Income Countries | |
| **Females** | | | | | | | | |
| Intercept | **9.24 (.03)** | **12.45 (.03)** | **10.05 (.03)** | **8.76 (.03)** | **19.28 (.04)** | | **9.27 (.04)** | |
| Linear slope | - | - | - | **0.57 (.01)** | -.58 (.02) | | .13 (.02) | |
| Chi-square likelihood ratio tests | | | | | | | | |
| AIC | 2080.3 | | | 2010.9 | | | | |
| BIC | 2156.3 | | | 2125.0 | | | | |
| P (> Chisq) |  | | | **<.001** | | | | |
| **Males** | | | | | | | | |
| Intercept | **11.03 (.02)** | **13.69 (.03)** | **12.12 (.03)** | **8.58 (.03)** | | **7.90 (.04)** | | **17.36 (.04)** |
| Linear slope | - | - | - | **0.22 (.01)** | | -.22 (.02) | | 0.27 (.02) |
| Chi-square likelihood ratio tests | | | | | | | | |
| AIC | 2015.6 | | | 1989.2 | | | | |
| BIC | 2091.0 | | | 2102.2 | | | | |
| P (> Chisq) |  | | | **<.001** | | | | |

*Note.* The table reports the iterative series of multigroup latent growth curve models performed to examine how future orientation changes over time for both females and males. The first model refers to an intercept-only model where future orientation was allowed to vary in the first wave of data collection but not over time (therefore not including slope). The second model refers to a linear model where we assumed a constant change in future orientation over time (therefore including intercept and linear slope). Third and fourth models (i.e., quadratic and cubic models) were also tested, but they are not reported as they led to estimation issues. In the table, standardized estimates are reported, and bold values refer to *p* < .05. The symbol – indicates that the form (i.e., slopes) was not tested in the model. Values represent unstandardized regression coefficients (*β*) with standard errors (SE) in parentheses, AIC = Akaike information criterion; BIC = Bayesian information criterion; *p* (> χ²) = probability associated with the chi-square test.

**Supplemental Table 4**

*Conditional Model Comparisons using Chi-square Likelihood Ratio Tests for Females Across Parenting Dimensions and Parent Gender by Country Income Level*

|  | Parental Monitoring (M) | Parental Monitoring (F) | Family Obligations  (M) | Family Obligations  (F) | Individualism  (M) | Individualism  (F) | Collectivism  (M) | Collectivism  (F) | Conformity Values  (M) | Conformity Values  (F) |
| --- | --- | --- | --- | --- | --- | --- | --- | --- | --- | --- |
|  | β (SE) | β (SE) | β (SE) | β (SE) | β (SE) | β (SE) | β (SE) | β (SE) | β (SE) | β (SE) |
|  | **High Income Countries** | | | | | | | | | |
| Intercept | .04 (.06) | -.04 (.05) | .03 (.06) | **-.35 (.09)** | -.04 (.08) | -0.17 (.10) | **.23 (.09)** | **.32 (.12)** | .12 (.02) | .01 (.04) |
| Linear slope | .03 (.04) | .24 (.03) | -.09 (.03) | .13 (.04) | -.06 (.03) | .18 (.05) | -.14 (.05) | **-.45 (.06)** | **-.25 (.01)** | .02 (.02) |
|  | **Upper-Middle Income Countries** | | | | | | | | | |
| Intercept | .46 (.06) | -.16 (.06) | .15 (.08) | **-1.04 (.09)** | .11 (.12) | -.16 (.12) | .43 (.13) | .02 (.10) | **.64 (.03)** | .09 (.03) |
| Linear slope | -.16 (.02) | .43 (.03) | -.03 (.05) | **.78 (.04)** | **-.69 (.06)** | .56 (.07) | -.37 (.07) | -.49 (.06) | -.01 (.01) | -.08 (.01) |
|  | **Lower-Middle Income Countries** | | | | | | | | | |
| Intercept | **-.24 (.07)** | **.28 (.06)** | -.24 (.08) | .26 (.08) | -.02 (.10) | -.14 (.09) | .16 (.10) | -.12 (.09) | -.07 (.03) | -.08 (.03) |
| Linear slope | .08 (.03) | -.08 (.03) | .26 (.04) | **-.35 (.04)** | -.11 (.05) | .04 (.04) | **-.31 (.04)** | **.30 (.05)** | .03 (.02) | .11 (.01) |

*Note.* In the table, standardized estimates are reported, and bold values refer to *p* < .05. M = mother; F = father. Values represent unstandardized regression coefficients (*β*) with standard errors (SE) in parentheses.

**Supplemental Table 5**

*Conditional Model Comparisons using Chi-square Likelihood Ratio Tests for Males Across Parenting Dimensions and Parent Gender by Country Income Level*

|  | Parental Monitoring (M) | Parental Monitoring (F) | Family Obligations  (M) | Family Obligations  (F) | Individualism  (M) | Individualism  (F) | Collectivism  (M) | Collectivism  (F) | Conformity Values  (M) | Conformity Values  (F) |
| --- | --- | --- | --- | --- | --- | --- | --- | --- | --- | --- |
|  | B (SE) | B (SE) | B (SE) | B (SE) | B (SE) | B (SE) | B (SE) | B (SE) | B (SE) | B (SE) |
|  | **High Income Countries** | | | | | | | | | |
| Intercept | -.02 (.05) | .02 (.04) | .05 (.05) | -.00 (.05) | .02 (.04) | .01 (.05) | -.00 (.07) | **.22 (.08)** | -.01 (.02) | **.04 (.02)** |
| Linear slope | -.01 (.02) | -.01 (.02) | .00 (.03) | -.00 (.03) | -.04 (.03) | -.01 (.03) | .03 (.03) | **-.08 (.04)** | -.01 (.01) | **-.02 (.01)** |
|  | **Upper-Middle Income Countries** | | | | | | | | | |
| Intercept | -.02 (.05) | .02 (.04) | .05 (.05) | -.00 (.05) | .02 (.04) | .01 (.05) | -.00 (.07) | **.22 (.08)** | -.01 (.02) | **.04 (.02)** |
| Linear slope | -.01 (.02) | -.01 (.02) | .00 (.03) | -.00 (.03) | -.04 (.03) | -.01 (.03) | .03 (.03) | **-.08 (.04)** | -.01 (.01) | **-.02 (.01)** |
|  | **Lower-Middle Income Countries** | | | | | | | | | |
| Intercept | -.02 (.05) | .02 (.04) | .05 (.05) | -.00 (.05) | .02 (.04) | .01 (.05) | -.00 (.07) | **.22 (.08)** | -.01 (.02) | **.04 (.02)** |
| Linear slope | -.01 (.02) | -.01 (.02) | .00 (.03) | -.00 (.03) | -.04 (.03) | -.01 (.03) | .03 (.03) | **-.08 (.04)** | -.01 (.01) | **-.02 (.01)** |

*Note.* In the table, unstandardized estimates are reported, and bold values refer to *p* < .05. M = mother; F = father. Values represent unstandardized regression coefficients (*β*) with standard errors (SE) in parentheses. The unstandardized estimates are reported as the estimates were constrained to be equal across groups.
